# Dental expenditure, progressivity and horizontal inequality in Chinese adults: based on the 4th National Oral Health Epidemiology Survey

**DOI:** 10.1186/s12903-020-01128-0

**Published:** 2020-05-11

**Authors:** Meng Lin Cheng, Chun Xiao Wang, Xing Wang, Xi Ping Feng, Bao Jun Tai, Yu De Hu, Huan Cai Lin, Bo Wang, Shu Guo Zheng, Xue Nan Liu, Wen Sheng Rong, Wei Jian Wang, Yan Si, Tao Xu

**Affiliations:** 1 Department of Stomatology, Beijing Friendship Hospital, Capital Medical University, Beijing, China; 2 Center for Chronic and Non-communicable Disease Control and Prevention, Chinese Center for Disease Control and Prevention, Beijing, China; 3 National Engineering Laboratory for Digital and Material Technology of Stomatology, Beijing Key Laboratory of Digital Stomatology, Chinese Stomatological Association, Peking University School and Hospital of Stomatology, Beijing, China; 4 Department of Preventive Dentistry, Shanghai Ninth People’s Hospital, Shanghai Jiao Tong University School of Medicine, Shanghai, China; 5 Department of Preventive Dentistry, the State Key Laboratory Breeding Base of Basic Science of Stomatology (Hubei-MOST) & Key Laboratory of Oral Biomedicine Ministry of Education, School & Hospital of Stomatology, Wuhan University, Wuhan, China; 6 Department of Preventive Dentistry, West China School of Stomatology, Sichuan University, Chengdu, China; 7 Department of Preventive Dentistry, Guanghua School of Stomatology, Hospital of Stomatology, Sun Yet-sen University, Guangzhou, China; 8 Guangdong Provincial Key Laboratory of Stomatology, Guangzhou, China; 9 Department of Preventive Dentistry, Peking University School and Hospital of Stomatology, National Engineering Laboratory for Digital and Material Technology of Stomatology, Beijing Key Laboratory of Digital Stomatology, Beijing, China

**Keywords:** Dental expenditure, Horizontal inequality, Kakwani index, Decomposition of concentration index, Socioeconomic determinants

## Abstract

**Background:**

The financial burden of oral diseases is a growing concern as the medical expenses rise worldwide. The aim of this study was to investigate the dental expenditure, analyze its progressivity and horizontal inequality under the general health finance and insurance system, and identify the key social determinants of the inequality for Chinese adults.

**Methods:**

A secondary analysis used the data of 13,464 adults from the 4th National Oral Health Epidemiological Survey (NOHES) in China was undertaken. The dental expenditure was collected and divided into out-of-pocket and health insurance payments. Horizontal inequality index and Kakwani index were used to analyze the horizontal inequality and progressivity, respectively. The decomposition model of the concentration index was set up to explore the associated socioeconomic determinants.

**Results:**

The results showed that a mean dental expenditure per capita of Chinese adults was $20.55 (95% Confidence Interval-CI: 18.83,22.26). Among those who actually used dental service, the cost was $100.95 (95%CI: 93.22,108.68). Over 90% of dental spending was due to out-of-pocket expenses. For self-reported oral health, the horizontal inequality index was − 0.1391 and for the decayed tooth (DT), it was − 0.2252. For out-of-pocket payment, the Kakwani index was − 0.3154 and for health insurance payment it was − 0.1598. Income, residential location, educational attainment, oral hygiene practice, self-reported oral health, age difference were the main contributors to the inequality of dental expenditure.

**Conclusion:**

Dental expenditure for Chinese adults was at a lower level due to underutilization. The ratio of payments of dental expenditure and utilization was disproportional, whether it was out-of-pocket or insurance payment. Individuals who were more in need of oral care showed less demand for service or not required service in time. For future policy making on oral health, it is worth the effort to further promote the awareness of the importance of oral health and utilization of dental service.

## Background

The financial burden of oral diseases is a growing concern as the medical fee rise worldwide. The World Health Organization (WHO) reported that the treatment of oral diseases was the 4th expense in most industrial countries [1]. In the latest research on the global burden of diseases, oral diseases affect the lives of 3.5 billion people worldwide and become a global public challenge [2, 3]. An up-to-date economic estimation claimed that direct treatment costs due to dental diseases worldwide were estimated at 298 billion US dollars (USD) yearly, corresponding to an average of 4.6% of global health expenditure [4]. Another study demonstrated that severe teeth loss was found to imply 67% of losses of global productivity, followed by severe periodontitis (21%) and untreated caries (12%) [5].

Significant inequalities exist in oral health, such as oral health condition, utilization of services and unbalanced expenditures distribution among populations. Low socioeconomic status was found associated with severe caries and less utilization of dental services [6, 7]. Social and demographic factors affect the use of dental services, both directly and through insurance participation [8]. Income inequality is a potential influence in both social status and utilization of oral health [9]. Dentistry is often unaffordable and/or unavailable, particularly for those in the poor rural areas in the low- or middle-income countries [10].

From 2009, the Chinese government deepened the reform of the medical health care system in which the basic medical health insurance structurally covered 90% of people [11]. However, most of them are not covered for oral diseases and over 85% of dental expenditure are out-of-pocket payments [12]. It is imperative to further analyze and improve the current situation in order to provide equality in health care including oral health.

In the previous NOHES in China, the income-related inequality in oral health was not evaluated [13]. The 4th NOHES conducted in 2015–2016 firstly surveyed this subject to provide information for the future development of oral health-related policies.

This study mainly used the data from the 4th NOHES for secondary analysis aiming to describe the dental expenditure, analyze its progressivity and horizontal inequality, and identify the relevant social determinants for oral health for Chinese adults such as income or health insurance.

## Methods

### Data sources

The 4th NOHES in China was a pathfinding survey used a multistage, random, stratified, equal volume sampling method. Groups of 35–44 years old and 65–74 years old adults were selected representing young adults and the elderly under the WHO guideline. A 55–65 years old group was additionally investigated to know more about the middle-aged. All 13,464 participants were included as a representative sample of Chinese adults. The detailed sampling methods can be found in the series of publications [14]. Based on the 5th edition of the WHO Oral Health Survey [15], oral health examination and oral health-related questionnaires were conducted. The 6th Census statistics data from the National Bureau of Statistics online [16] was used to computed the weight based on the sample’s province, residential location (urban or rural area), age and gender in order to obtain an unbiased estimation [17, 18].

Ethical approval (Approval No: 2014–003) for the study was received from the Ethics Committee of Chinese Stomatological Association and informed consent was obtained.

### Expenditure estimation and distribution of health insurance

The questions for dental expenditure and out-of-pocket payment were given as “How much have you paid for a dental visit last year?” and “What was the self-paid ratio in the above dental expenditure?” The health insurance payment was calculated as the difference between total expenditure and out-of-pocket payment. Information like household income and expenditure was avoided because of privacy concerns, the demographic and socioeconomic characteristics of these 15.5% participants who did not report the two key information were unbalanced with the total population, missing values of key variables were filled by medians. Dental expenditure incurred when participants used dental service. Only 2740 people of the total 13,464 participants used dental services in the past year and the expenditures of these two populations were both estimated. The expenditure was converted according to the 2016 Chinese Yuan (RMB) to the USD exchange rate that 100USD was equivalent to 664.23RMB. According to the data from the National Bureau of Statistics, the medical expenditure per capita was equivalent to 504.61USD (http://data.stats.gov.cn/easyquery.htm?cn=C01&zb=A0O0K&sj=2016) and the dental expenditure per capita as a proportion of it was roughly calculated.

The basic medical insurance consists of urban employee basic medical insurance (UEBMI), urban resident basic medical insurance (URBMI) and new rural cooperative medical care (NRCMC). Other health insurances include government medical insurance for government officials and private commercial health insurance, accounting for a small proportion. The UEBMI is covered by urban employees and has the highest reimbursement ratio and the highest paid premiums. The URBMI and the NRCMC are covered by urban and rural residents based on household registration, respectively. The URBMI has higher reimbursement in hospitalization and outpatient treatment for severe diseases than NRCMC. Among the public health insurance, many basic oral therapeutic services have been included or adjusted in the catalog of basic medical insurance. However, the thresholds and reimbursement for different types of insurance are different.

### Horizontal inequality and progressivity

The inequality can be assessed through variation in interesting variables such as health needs, medical services and expenditure across quintiles of income [19]. In the household income quintiles figure, participants are sorted by household income from poor to rich and divided evenly into five groups. As income increase, the change of expenditure, oral health need, and dental service indicated whether inequality exists. And this figure will give an intuitive, qualitative description of inequality. Besides, a complete picture can be drawn by concentration curves. And the associated horizontal inequality index and Kakwani index are described as follows:

The horizontal equality indicates that people with equal health need to obtain equal medical care [20]. When the index is negative and the concentration curve of medical need is above the concentration curve of medical care, the inequality is in favor of the rich [21]. In this case, poor people with more medical needs receive less medical care. The formula of horizontal inequality is:

$$ HI={C}_M-{C}_N $$

The *C*_*M*_ and the *C*_*N*_ are the concentration indices for medical care and need, respectively. The formula of them are:

$$ {C}_M=1-2{\int}_0^1 Lu(x) dx $$

$$ {C}_N=1-2{\int}_0^1 Ln(x) dx $$

When people are sorted by the variable of ability to pay (ATP) from poor to rich, the cumulative proportion of utilization graphs the concentration curve of medical care (*Lu*). As the utilization of dental service is treatment-oriented in China [22] d based on the behavior model of Anderson [23], an evaluated health variable for objective needs and the other subjective ones which reflect the demand for dental care should be considered at the evaluation of *C*_*N*_. Consider DT (Decayed teeth) reflected the unsatisfied primary dental need, it is used to be an evaluated need variable. Correspondingly, the self-reported oral health status represented the subjective medical need. The self-reported oral health was an ordered five categorical variables in the questionnaire and higher ratings indicate worse self-perceived oral health. The two variables form two concentration curves for medical needs (*Ln*). The HI is twice the area of curves between the *Lu* and the *Ln* and rank from − 1 to 1.

The vertical equality refers to a balanced proportion of medical expenses against people’s ATP. A progressive payment route means the extent to which medical expenditure rise as a proportion of people’s ATP when his or her ATP rises. If it is converse, the payment is regressive. If people with different income levels have the same ratio of their medical expenses to their ATP, it is believed that the funding is balanced [21].

The Kakwani index is used most widely for progressivity research [24]. The Kakwani index is defined as the difference between concentration index (*Cp*) and Gini coefficient(*G*), in other words, it is twice the area between the concentration curve of payment route (*Lp*) and the Lorenz curve of ATP (*Lg)*. In this study, household income was used to represent the ATP.

The formulas of *Cp, G* and Kakwani index (*π*_*k*_) are as follow:

$$ Cp=1-2{\int}_0^1 Lp(x) dx $$

$$ G=1-2{\int}_0^1 Lg(x) dx $$

$$ {\pi}_k= Cp-G $$

Similar to the HI, people are sorted by household income from low to high and the cumulative proportion of dental expenditure in different payment routes or household income is graphed. *Cp* ranks from − 1 to 1 and the value of Gini coefficient ranges from 0 to 1 [25]. As the dental expenditure concentrated in the population who used dental service in the past year, based on the consideration of different socioeconomic characteristics distribution, the Kakwani indices of total participants and those who used dental service in the past year were calculated and compared to strengthen the validity of results. For HI or Kakwani index, the positive or negative of the index indicates that the inequality is concentrated in the rich or the poor, and the magnitude of the value reflects the degree of such inequality.

### Decomposition of concentration indices

Two models were constructed to decompose the contributions of the dental expenditure concentration index and the first of them enrolled all participants while the second one enrolled only participants used dental service in the past year. It was decomposed into four sources: (i) socioeconomic characteristics which consist of household income, region, residential location, education attainment and the coverage of different types of health insurance;(ii) the need variables which include evaluated need (DT) and subjective need (the level of self-reported oral health status), and oral health behavior (teeth brushing habit); (iii) socio-demographic characteristics which include age groups, gender, nationality;(iv) medical care variable which refers specifically to the utilization of dental service in the past year only in the first model. According to the nonlinear model, the concentration index for dental expenditure (*Cp*) can be decomposed as:

$$ Cp=\sum \left({\upbeta}_{\mathrm{k}}{\mathrm{x}}_{\mathrm{k}}/\upmu \right){\mathrm{C}}_{\mathrm{k}}+\mathrm{GC}\upvarepsilon /\upmu $$

Where μ is the mean of dental expenditure, C_k_ is the concentration index for x_k_ variable and x_k_ is the mean of x_k_, GCε is the generalized concentration index for the error term(ε). The *Cp* is equal to a weighted sum of the concentration indices of the k variables, where the β _k_x_k_/ μ indicates elasticity of for x_k_.

SPSS 22.0 was used to be the data processing software to deal with the distribution of dental expenditure and health insurance. STATA 14.0 was used to drew the figures of inequalities and set up the decomposition models. The code for decomposition was referenced in the guide of health equity analysis [24].

## Results

**Fig. 1:**
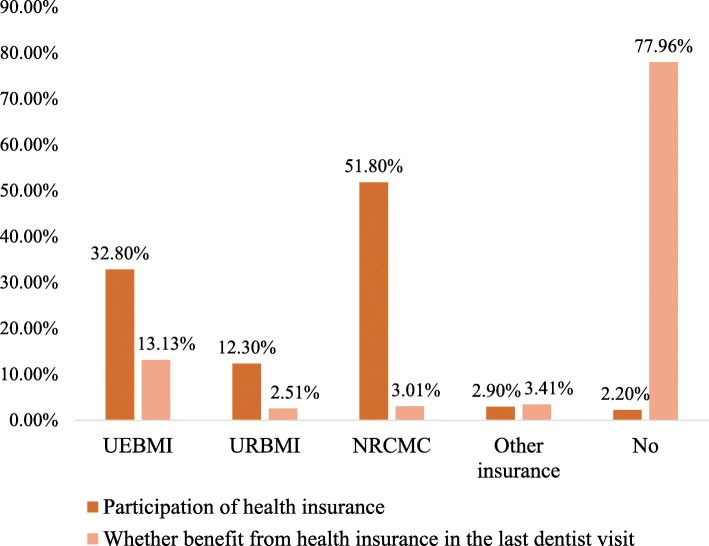
Distribution of different types of health insurance. Comparison between participation of health insurance in all participants (*n* = 13,464) and whether participants who used dental service in the past year (*n* = 2740) benefit from these insurance in the last dentist visit. Other insurances included government insurance and private insurance and they were not conflict to the basic medical health insurance system

The total dental expenditure per capita was $20.55(95%CI: 18.83,22.26) for all participants and $100.95(95%CI, 93.22,108.68) for those who used dental service in the past year. More than 90% of dental expenditure was paid out-of-pocket. The dental expenditure per capita accounted for approximately 4.08% of the total medical expenses per capita. In Fig. 1, 96.9% of the 13,464 participants were enrolled in the basic health insurance, only 2.2% participants did not register in any insurance. However, 77.8% of 2740 who used dental service in the past year reported they paid out-of-pocket for dental service but among these participants, only 2.6% were not enrolled in medical health insurance.

**Fig. 2:**
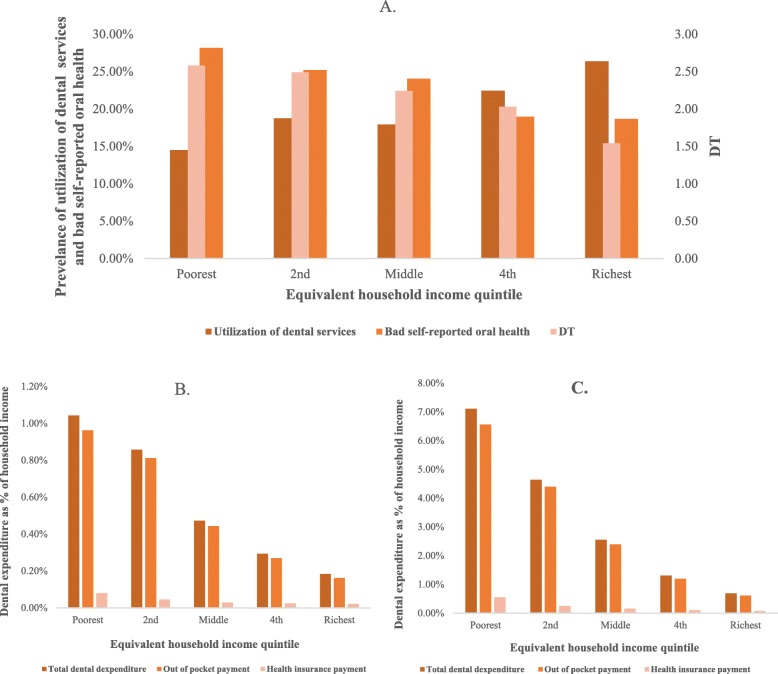
Dental expenditure, dental care and dental needs in different household income groups. **a** Different distributions of dental care and needs in household income groups from poor to rich. The utilization of dental service in the past year indicated the dental care, the DT and the bad self-reported oral health indicated evaluated and subjective dental needs, respectively. **b** Different payments routes as percentage of household income for all participants—averaged by household income quintile. **c** Different payment routes as percentage of household income for those who used dental services in the past year—averaged by household income quintile

The household income quintile bar charts (Fig. 2) showed the trends of dental expenditure, need and service utilization as income level rise. For horizontal analysis, utilization of dental service increased and dental need decreased as the household income level increased. The quintile with the highest prevalence of bad self-reported oral health obtained less utilization of dental service. At the same time, the quintile with less DT acquired more utilization of dental service. The proportion of dental expenditure in household income declined as household income level raised. For the poorest quintile in those who used dental service in the past year, such proportion was more than 7% but for the richest, this number was less than 1%.

**Fig. 3:**
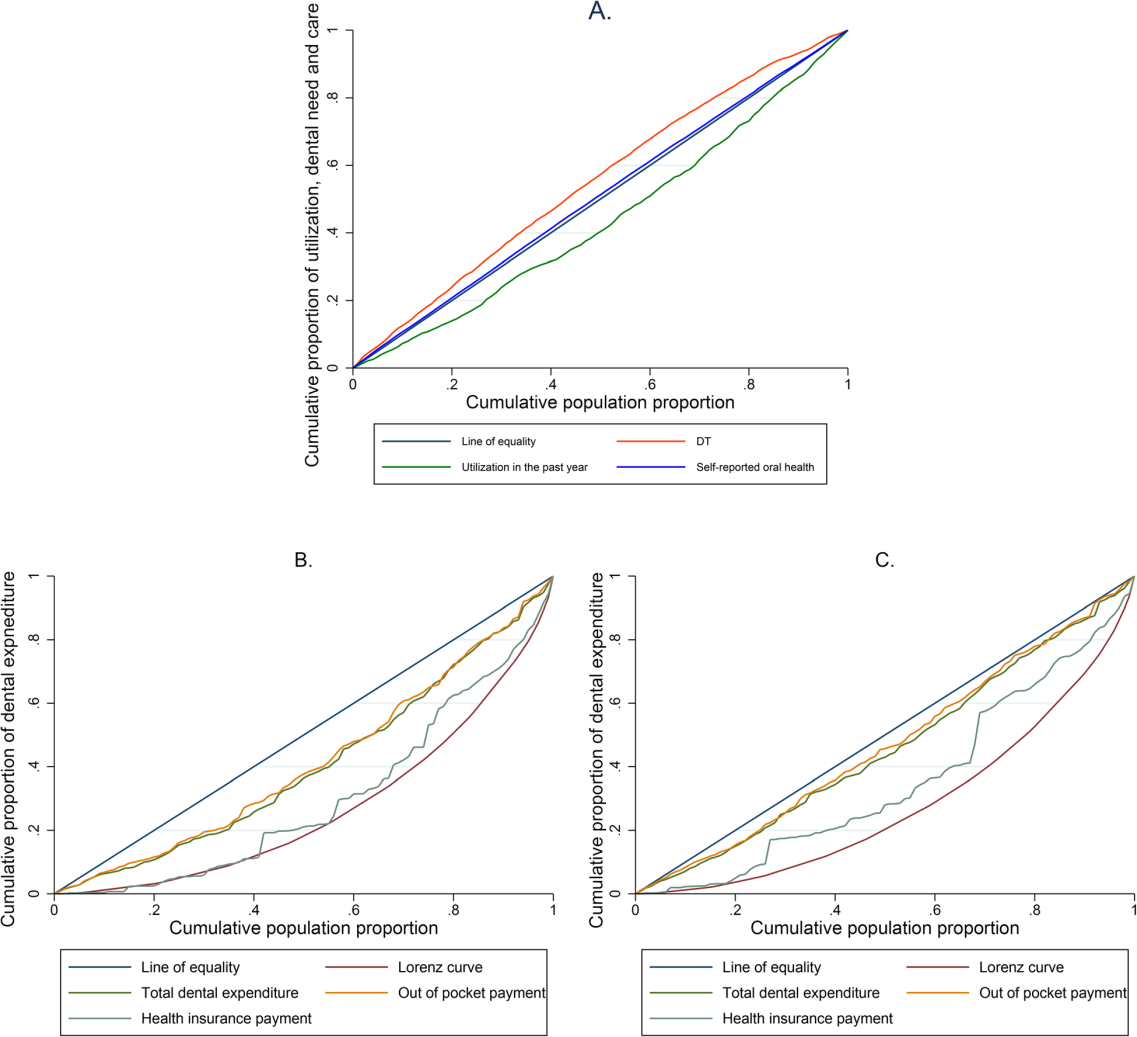
Concentration curves and Lorenz curve for dental expenditure, dental care and dental needs. **a** Concentration curves for dental need and care. DT and self-reported oral health were variable referred to evaluated and subjective dental need, respectively. Dental services utilization in the past year referred to the situation of dental care. **b** Concentration curves for different payment routes and Lorenz curve for household income in all participants. **c** Concentration curves for different payment routes and Lorenz curve for household income in those who used dental services in the past year

The analysis results showed in Table 1 also demonstrated the inequality in dental expenditure. And the concentration curves showed in Fig. 3 were consistent with such results. For self-reported oral health, the horizontal inequality index was − 0.1391 and for decayed tooth (DT), it was − 0.2252. For out-of-pocket payment, the Kakwani index was − 0.3154 and for health insurance payment it was − 0.1598. The Kakwani indices and Horizontal inequality indices were negative and statistically significant. Medical care was in favor of the rich but medical need was concentrated in the poor. However, the distribution of self-reported oral health trended to be more balanced. People’s assessment to their oral health was more optimistic. Out-of-pocket payments and health insurance payments both benefit the rich, but the former is more concentrated among the rich. In Fig. 3, the distribution of the total dental expenditures in both all participants and those who used dental service in the past year were similar to the out-of-pocket payment. The difference was that inequality appeared to be expanding among the population who used dental services.

**Table 1:** Shares of dental expenditure, utilization of dental service and dental need for all participants

Quintiles	Household income	Vertical inequality items			Horizontal inequality items		
		Total dental expenditure	Out-of-pocket payment	Health insurance payment	Utilization in the past year	Self-reported oral health	DT
Poorest	3.18%	10.99%	11.09%	9.93%	14.50%	24.50%	23.73%
2nd	4.42%	12.59%	13.03%	7.86%	18.74%	21.89%	22.90%
Middle	11.78%	18.45%	18.92%	13.41%	17.94%	20.89%	20.55%
4th	24.16%	23.53%	23.62%	22.65%	22.43%	16.49%	18.67%
Richest	56.46%	34.44%	33.35%	46.15%	26.39%	16.23%	14.16%
Concentration index/Gini coefficient	0.4974	0.1952	0.182	0.3376	0.1215	−0.0176	− 0.1036
(standard error)	−0.0039	− 0.0309	− 0.0325	− 0.0621	− 0.0128	−0.0021	0.0088
(*p* value)	(< 0.001)	(< 0.001)	(< 0.001)	(< 0.001)	(< 0.001)	(< 0.001)	(< 0.001)
Kakwani index/	/	−0.3022	−0.3154	− 0.1598	/	− 0.1391	− 0.2252
Horizontal inequality index							
(standard error)	/	−0.031	−0.0327	− 0.0621	/	− 0.0127	0.0152
(p value)	/	(< 0.001)	(< 0.001)	−0.010	/	(< 0.001)	(< 0.001)

Legend: All participants were sorted by household income from poor to rich and evenly divided into five groups. The proportion of interested variables of each group against the whole participants were recorded. Proportion for ‘self-reported oral health’ here referred to proportion of poor and very poor self-reported oral health population. Household income was the ranking and reference variable that referred to the ability to pay

The results of the two decomposition models were consistent in Table 2, which showed the reliability of the inclusion factors. Undoubtedly, utilization was the most important contributor. Socioeconomic characteristics, including household income, residential location and education attainment, gave most contribution to the disparity of dental expenditure. Besides, good oral hygiene practice contributed a lot to this inequality. People with good oral hygiene was more concentrated in the rich. As for age, dental expenditure was more in favor of old people. Other demographic characteristics like gender or nationality did not contribute a lot. Compared to the evaluated need (DT), the subjective need (self-reported oral health) contributed more. The contribution of dental need was negative as bad self-reported oral health were concentrated in the poor. Health insurance except the URBMI did not give definite contributions in Model 1 but the NRCMC gave a larger opposite contribution in Model 2.

**Table 2:** Decomposition of concentration index for dental expenditure

Variables	Model 1				Model 2			
	Elasticities	Concentration indices	Contributions	Percentage of contributions	Elasticities	Concentration indices	Contributions	Percentage of contributions
Household income	0.0716	0.4975	0.0356	18.24%	0.0715	0.4757	0.0340	50.43%
Central region	−0.0838	−0.0565	0.0047	2.42%	−0.0811	−0.0312	0.0025	3.76%
Western region	−0.0166	−0.0884	0.0015	0.75%	−0.0141	−0.1092	0.0015	2.29%
Area-Urban area	−0.2886	−0.0554	0.0160	8.19%	−0.3363	−0.0441	0.0148	22.02%
Education	0.1227	0.1138	0.0140	7.15%	0.1575	0.0919	0.0145	21.48%
UEBMI	0.0109	0.2668	0.0029	1.49%	0.0413	0.1945	0.0080	11.92%
URBMI	−0.0064	0.0520	−0.0003	−0.17%	0.0007	−0.0071	0.0000	−0.01%
NRCMC	0.0036	−0.1825	−0.0007	− 0.34%	0.0215	− 0.2233	−0.0048	−7.12%
Other insurance	0.0162	0.3166	0.0051	2.63%	0.0122	0.2540	0.0031	4.60%
Age	0.3584	−0.0466	−0.0167	−8.56%	0.3578	−0.0373	−0.0133	−19.79%
Gender-Female	0.0707	−0.0125	−0.0009	− 0.45%	0.0904	− 0.0171	−0.0015	−2.30%
Nationality-Han	−0.0056	−0.0688	0.0004	0.20%	−0.0043	−0.2011	0.0009	1.27%
Teeth brushing habit-twice daily	0.0941	0.1494	0.0141	7.20%	0.1053	0.0995	0.0105	15.53%
Self-reported oral health	0.3398	−0.0176	−0.0060	−3.06%	0.4806	−0.0187	−0.0090	−13.32%
DT	−0.0062	−0.1036	0.0006	0.33%	−0.0002	−0.0914	0.0000	0.03%
Dental utilization	0.9682	0.1216	0.1177	60.28%	–	–	–	–
Residual			0.0072	3.70%			0.0062	9.22%
Total			0.1952	100.00%			0.0674	100.00%

Legend: Model 1 enrolled all participants and Model 2 enrolled those who used dental services in the past year

The reference of central region and western region was eastern region, eastern region had higher economic development level

Nouns after “-” for variables indicated the references for binary variables

The UEBMI indicated urban employee basic medical insurance; the URBMI indicated urban resident basic medical insurance; the NRCMC indicated new rural cooperated medical care; other insurance included government medical insurance and private commercial insurance; they were binary variables in the decomposition and the reference was didn’t covered by such insurance

## Discussion

In comparison to Japan, the dental expenditure per capita was $203 under public health insurance which covered approximately 70% and it accounted for 6.7% of total medical expenditure [26]. As for Italy, the dental expenditure per capita was nearly 250EUR and the public health care system only provided 5% of oral care [27]. In Australia, the mean total dental expenditure was 702 USD and mean out-of-pocket expenditure was 489 USD [28]. Dental expenditure for Chinese adults was at a lower level. The basic medical insurance for oral health did not change significantly compared to the previous study [8].

Two possible reasons could explain why nearly 80% of people used dental service but they did not get reimbursed from the insurance. One was that the reimbursable payment for dental service did not reach the threshold. One of the feedbacks of why not seeing a dentist, from a series of other studies in the 4th NOHES, was “financial difficulty” [29] . The threshold could be more than most people’s willingness to pay for dental services. The second was that the non-reimbursable portion was too much. People who might need prosthodontic, aesthetic and cosmetic dental services are not usually covered by insurance and the cost of service is expensive. Those were generally optimistic about their oral health, and the utilization of dental services is treatment-oriented. Preventive or regular dental visits will help solve the problem.

It is worth noticing that there is approximately 20% of utilization of dental service and there might be a huge space for growth in dental expenditure followed by economic advancement. Raising individual awareness of the importance of oral health should be valued and it will also be helpful for improving the utilization of dental service. Oral health education and promotion should continuously be the focus of public oral health implementations to shift to a preventive-initiated or regular-visit health care model.

The result from horizontal inequality analysis indicated financially disadvantaged individuals with more medical needs could not have full access to health services, which is a common problem in many countries around the world [21, 30]. Correspondingly, the bad self-reported oral health contributed a lot to the decomposition model. The Out-of-pocket and health insurance payments were both regressive and the out-of-pocket payment was more regressive than that of the insurance payment. The benefit from basic medical insurance was quietly limited. The polarization of medical spending in rich and poor indicated an inequality distribution of oral medical resources and could cause the polarization of oral health status [31–33]. After all, the extent to which patients have to pay for dental care and the manner in which dental care providers are reimbursed for their services have important bearings on the use and quality of care [10, 34].

The result from two decomposition models showed high consistency and reliability. Socioeconomic level directly contributed to the inequality of oral medical expenditure. The major contribution from household income, residential location and education attainment indicates the social class determined the inequality of dental expenditure. The positive contribution of teeth brushing habits means that good oral hygiene concentrated in the rich. In the three basic medical insurances, the contribution of the UEBMI was definite in both two models and the contribution of NRCMC seemed important in model 2. Combined with other descriptive results, it may only mean that the UEBMI had a higher capacity to share the financial risk of dental visits than the NRCMC.

The policy of comprehensively deepening medical reform in China has been implemented continuously but we cautiously think about that policy such as increasing the reimbursement ratio of basic medical insurance may not be effective for the equality of dental expenses because of the treatment-oriented utilization model remained unchanged. In the further oral health-related insurance system adjustment, the redistribution of medical expenditure through health insurance needs taking into account socioeconomic factors such as household income, residential location and education attainment.

### Limitation

For the first time, this study used a national epidemiological survey data to conduct an equality analysis of health financing for oral diseases. Biases in recall and report were unavoidable in such a cross-sectional survey. In this study, only the questions of the expenditure in the past year was answered to minimize potential recall bias. Besides, logically dental expenditure was made from utilization of dental service, the results of a full sample analysis could be diluted. Thus, one sample with all participants and the other with only those who used dental service in the past year were modeled and analyzed, respectively. The results from the two models showed consistency which supports the reliability of the study.

Based on the limitations of the survey data, the household income was used in this study to represent the ATP. In future research, variables such as wealth deposits and non-food expenditures and income may comprehensively reflect the ATP.

## Conclusion

Dental expenditure for Chinese adults was at a lower level due to the underutilization of dental service. The ratio of payments of dental expenditure and utilization was disproportional, regardless it was from out-of-pocket or insurance payment. Individuals who were more in need of oral care showed less demand for service or received dental services untimely. The service inequality was not in favor of low incomes. For future policy making, it is worth the effort to raise the public awareness of the importance of oral health and change the oral care model from treatment-oriented to preventive-initiated, and aid to set a habit for regular dental visits. If an oral health-related insurance system could be adjusted, socioeconomic status should be taken into account as it appears to be the main determinant of dental expenditure.

## Data Availability

The database of the 4th NOHES should not be shared publicly as it is a national database and the copyright does not allow. More relevant information about the NOHES can be provided in the official report [29]. The Census data is shared online as a reference [16].

## Abbreviations

ATP: Ability to pay
CI: Confidence Interval
DT: Decayed teeth
HI: horizontal inequality
NOHES: National Oral Health Epidemiology Survey
NRCMC: New Rural Cooperative Medical Care
RMB: Chinese Yuan
USD: US Dollar
UEBMI: Urban Employee Basic Medical Insurance
URBMI: Urban Residents Basic Medical Insurance
WHO: World Health Organization
